# Supplementary figures and images for: A high Mn(II)-tolerance strain, Bacillus thuringiensis HM7, isolated from manganese ore and its biosorption characteristics
Source: PeerJ. 2020 Feb 19;8:e8589. doi: 10.7717/peerj.8589 (PMC7363044; doi:10.7717/peerj.8589)

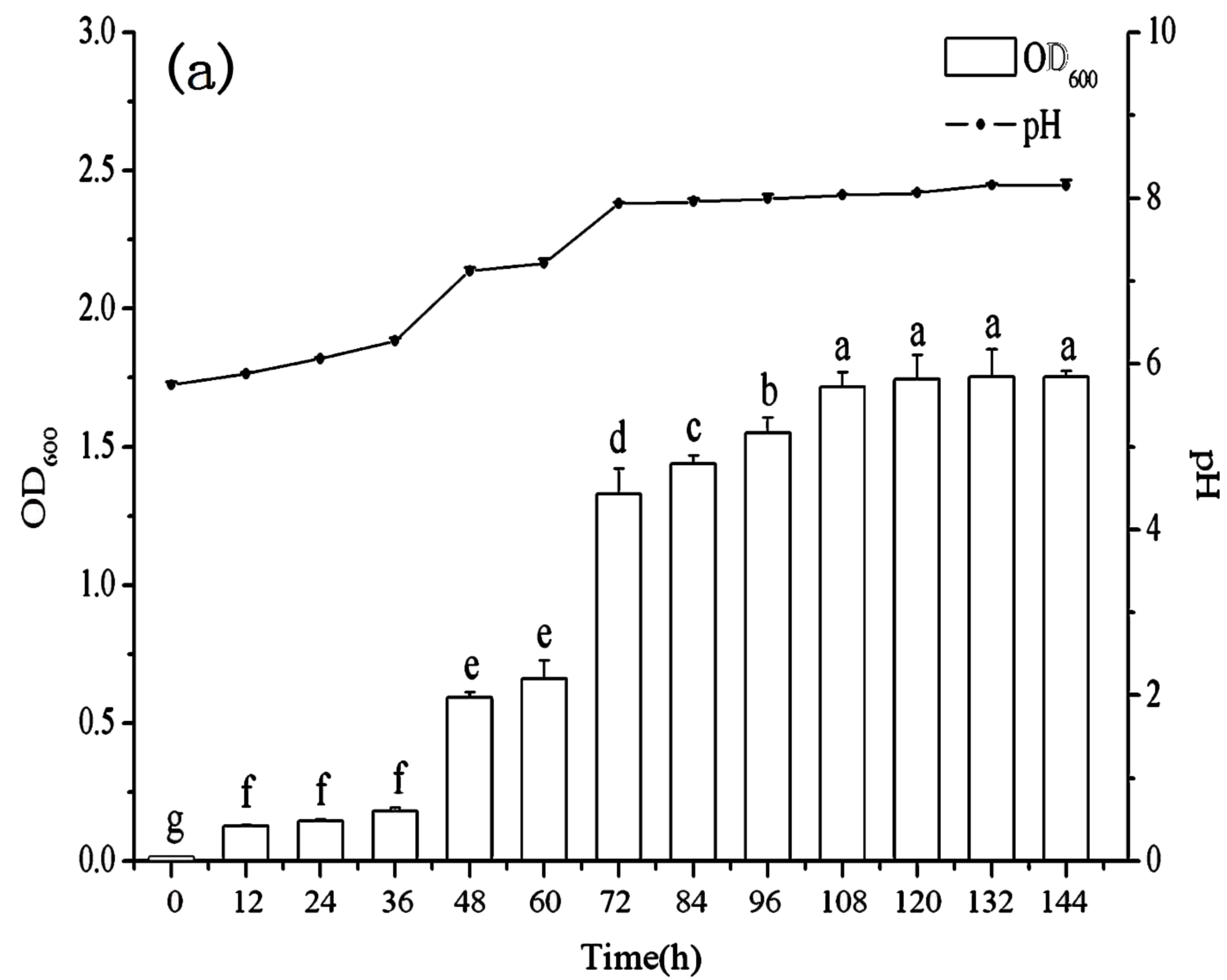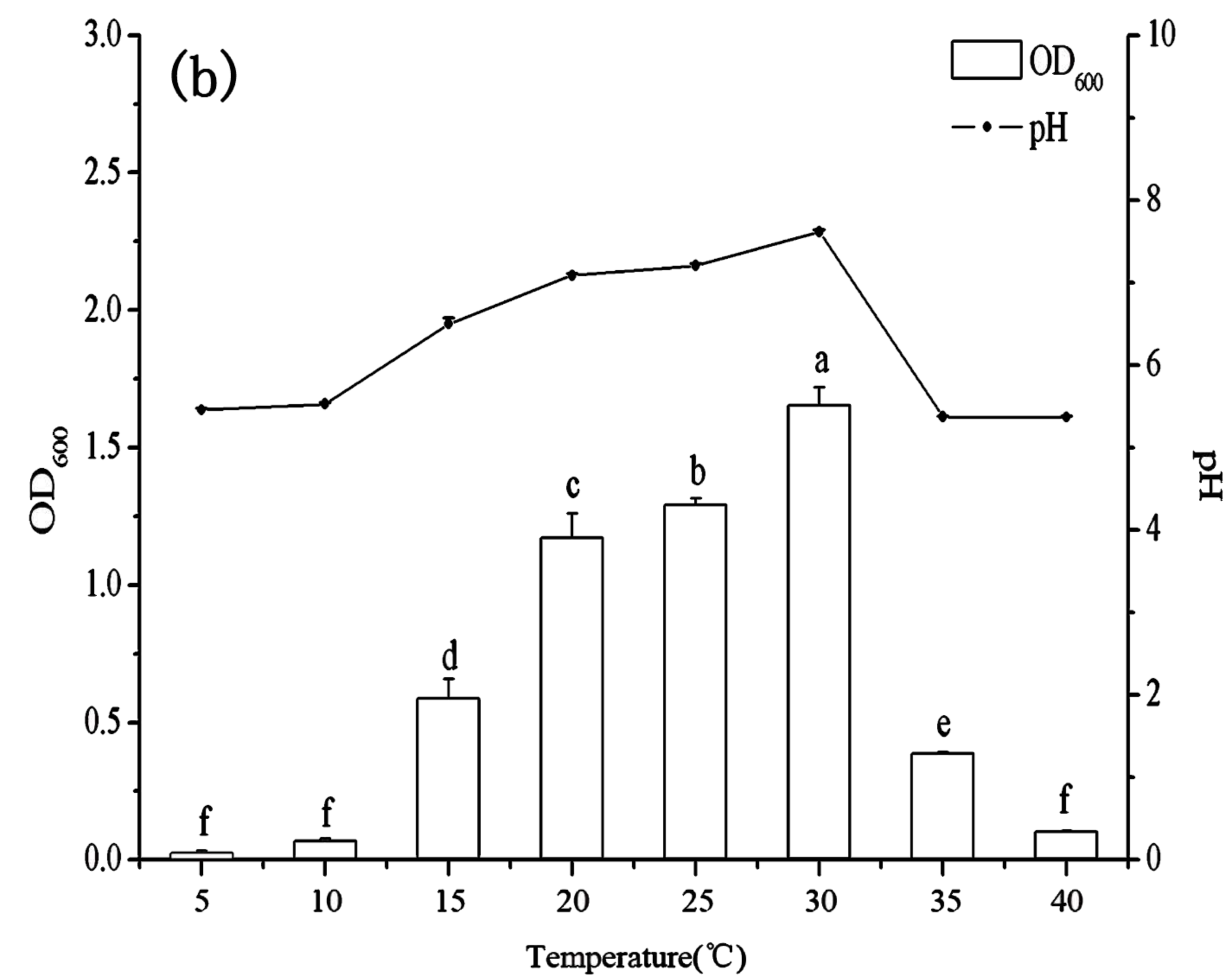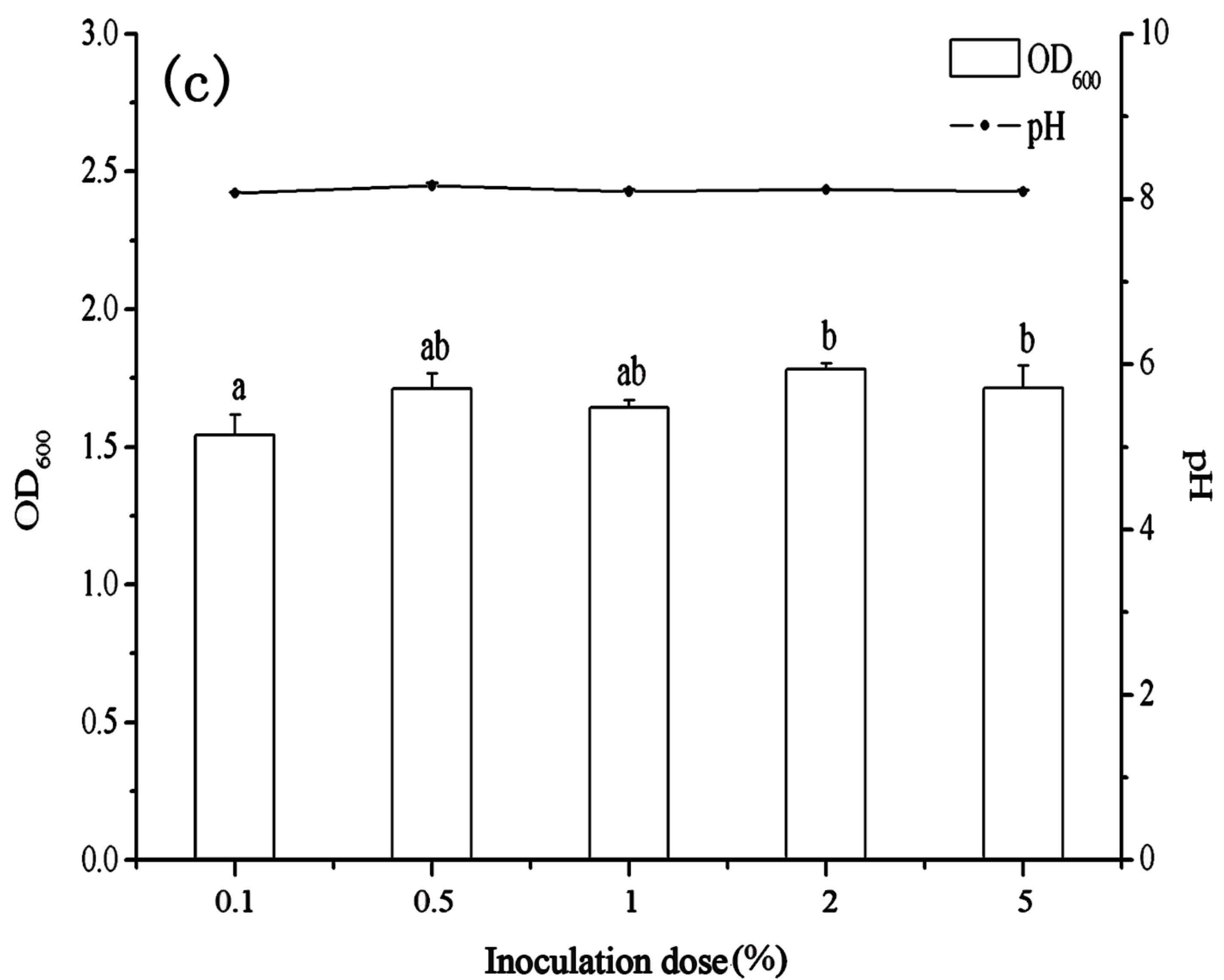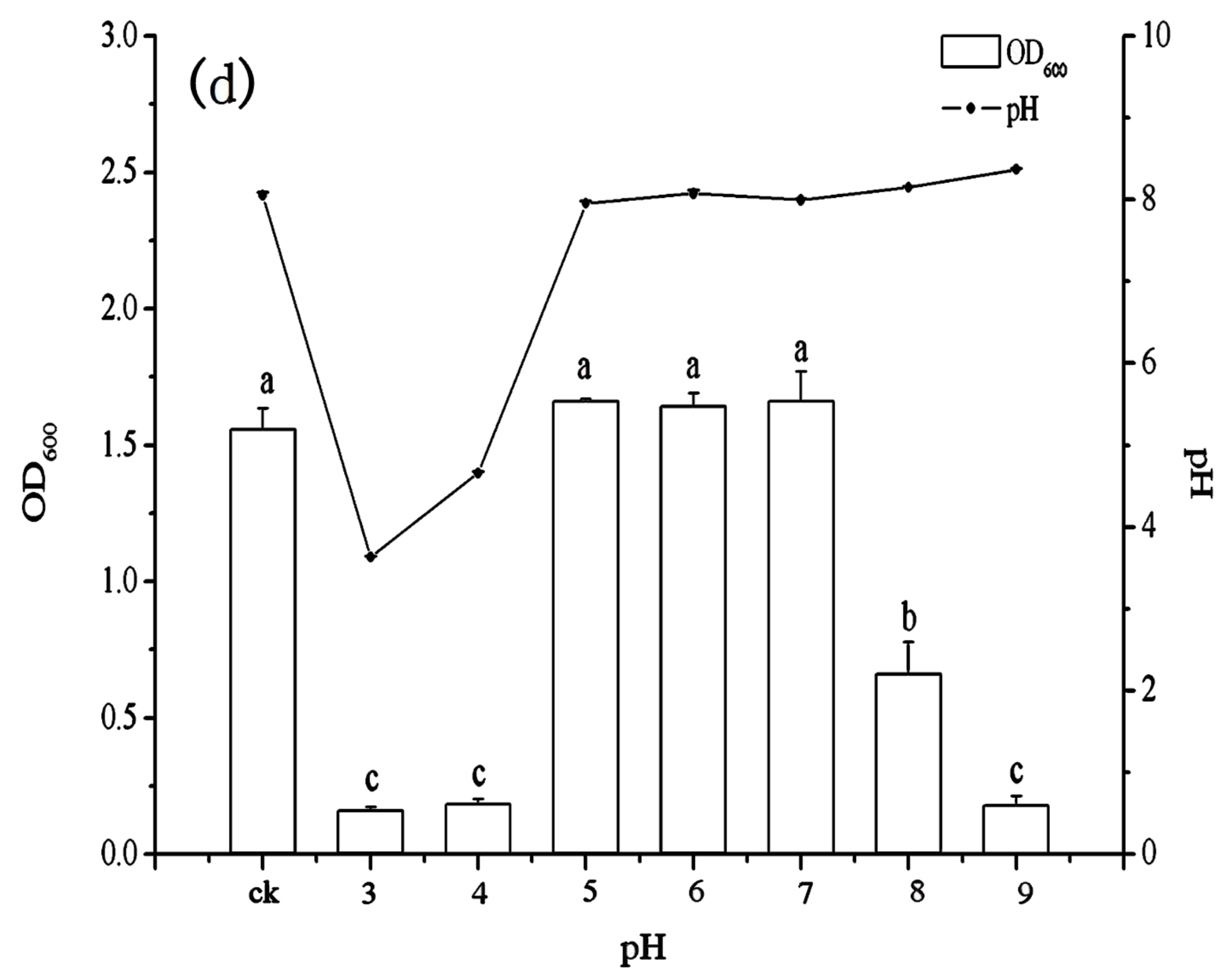

Supplement: Figure S1 — (a) at different time; (b) at different temperature; (c) at different inoculation dose; (d) at different pH (In general, the initial culture conditions were Mn(II) concentration 1,000 mg/L, temperature 30 °C and pH 5.6–5.8 for 72 h and change one of the corresponding factors under the single factor experiment). [file peerj-08-8589-s001.pdf]
