# Supplementary material for: A high Mn(II)-tolerance strain, Bacillus thuringiensis HM7, isolated from manganese ore and its biosorption characteristics
Source: PeerJ. 2020 Feb 19;8:e8589. doi: 10.7717/peerj.8589 (PMC7363044; doi:10.7717/peerj.8589)
Supplement: Supplemental Information 1 — Table S1: Characteristics of the B. thuringiensis HM7 in plant growth promoting and antibiotic resistance ability. Table S2: Comparison of Characteristics among the B. cereus HM5, B. thuringiensis HM7, and R. pickettii HM8 in plant growth promoting and antibiotic resistance ability. [file peerj-08-8589-s002.docx]

**Supplementary Table Legends**

Supplementary Table Comparison of Characteristics among the *B. cereus* HM5, *B. thuringiensis* HM7*,* and *R. pickettii* HM8 in plant growth promoting and antibiotic resistance ability

| Tests employed | HM5 | HM8 |
| --- | --- | --- |
| Removal amount (mg/L) | 593.36 | 1002.83 |
| **Plant growth promoting ability** |  |  |
| IAA (mg/L) | 2.36±0.02 ↓ | 1.70±0.04 ↓ |
| Phosphate Solubilization (mg/L) | 9.67±1.44 ↑ | 10.69±0.69 ↑ |
| Siderophores production | + / Y | - / N |
| **Antibiotic resistance** |  |  |
| Cefazolin (30 mcg) | N | Y |
| Amikacin (5 mcg) | Y | N |
| Chloramphenicol (30 mcg) | N | Y |
| Erythromycin (15 mcg) | N | Y |
| Trimethoprim (5 mcg) | N | N |
| Norfloxacin (10 mcg) | Y | N |
| Gentamycin (10 mcg) | Y | N |
| Ciprofloxacin (5 mcg) | N | N |
| Penicillin G(10 units) | N | N |
| Ampicillin (10 mcg) | N | N |

±, Standard deviation; +, Reaction; -, Without reaction; Y, Consistent with the results of HM7; N, Not consistent with the results of HM7; ↑, Value greater than HM7; ↓, Value less than HM7
